# Biocatalytic transamination in a monolithic flow reactor: improving enzyme grafting for enhanced performance†

**DOI:** 10.1039/c9ra02433f

**Published:** 2019-06-12

**Authors:** Ludivine van den Biggelaar, Patrice Soumillion, Damien P. Debecker

**Affiliations:** Institute of Condensed Matter and Nanosciences, UCLouvain Place Louis Pasteur 1 1348 Louvain-la-Neuve Belgium damien.debecker@uclouvain.be; Louvain Institute of Biomolecular Science and Technology, UCLouvain Place Croix du Sud 2 1348 Louvain-la-Neuve Belgium

## Abstract

Transaminases were immobilized onto macrocellular silica monoliths and used for carrying a continuous flow mode transamination reaction. Monoliths were prepared *via* an emulsion-templated sol–gel method and functionalised by amino-moieties (3-aminopropyl-triethoxysilane, APTES) in order to covalently immobilize the enzymes, using glutaraldehyde as a cross-linking agent. In order to obtain higher performance and improved reproducibility, we investigate the key parameters of APTES functionalisation and of enzyme grafting. Four functionalisation protocols were studied. We show that enhancing the homogeneity of the APTES grafting and controlling the moisture level during functionalisation led to a 3-fold increase in activity as compared to the previously reported data, and greatly improved the reproducibility. Additionally, we report a strong beneficial effect of running the enzyme immobilisation at room temperature instead of 4 °C, further enhancing the obtained activity. Finally, the popular method which consists of stabilizing the covalent attachment of the enzyme by reducing the imine bonds formed between the enzyme and the functionalised surface was investigated. We highlight a strong enzyme deactivation caused by cyanoborohydride, making this strategy irrelevant in this case. The improvements presented here led to more active macrocellular monoliths, of general interest for continuous flow mode biocatalysis.

## Abstract 2

Higher performance can be obtained in flow biocatalytic transamination reactions if the key parameters of support functionalization and of enzyme grafting are controlled.

## Graphical abstract

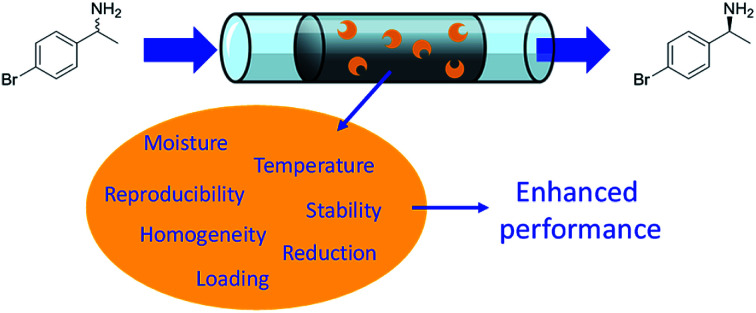

## Introduction

The use of enzymes for catalysing chemical transformations is of increasing interest, especially in the perspective of greener synthesis of high-value chemicals.^1–3^ Indeed, enzymes often work with high efficiency and (enantio)-selectivity, and in mild conditions.^4^ In this vibrant field of research, the biocatalytic synthesis of chiral amines using transaminase enzymes is a good example of an application for which tremendous achievements have been made, both at the fundamental and applied levels.^5–9^

In the perspective of practical applications, immobilizing enzymes on a solid carrier allows for their facile recovery (*e.g.* by filtration or centrifugation) so that the enzymes can then be reused several times.^10–12^ Among the various methods for enzyme immobilisation,^13^ covalent anchoring on porous carriers is probably the most successful approach, allowing a robust attachment of the enzyme.^11^ Importantly, the immobilisation of enzymes onto solid supports allows envisaging continuous flow biocatalytic processes to replace discontinuous batch processes.^14–16^ Such transfer from batch mode to continuous flow mode usually allows to increase the global productivity and to lower the environmental cost of chemical processes.^17–20^

Instead of the classical powdery materials that can be used as carriers and packed in a fixed bed reactor, a seducing approach for flow chemistry is to use self-standing porous monoliths.^21–23^ Inorganic or polymer monoliths containing intricate pore networks can be obtained in various desired shape and provide unique advantages such as fast kinetics and high throughput.^21^ Porous monolithic materials can have a high void fraction, thereby provoking only low pressure drop during the passage of a fluid.^24,25^

In particular, macrocellular silica monoliths have been extensively investigated because they can be obtained with tailored texture, structure, and surface functionalities, using bottom up sol–gel chemistry methods.^26–28^ In flow biocatalysis, for example, Brun *et al.*^29^ have reported on the use of macrocellular silica monoliths – coined “Si(HIPE)” – for the flow mode transesterification of crude oil using immobilized lipase. Si(HIPE) monoliths^27^ are obtained using a concentrated oil-in-water emulsion as a template. Their surface can then be functionalised, for example with epoxide or amine functions which serve as anchoring points for enzyme immobilisation.

Recently, we reported on the use of Si(HIPE) monoliths for the flow mode kinetic resolution of chiral amines using immobilized transaminases.^30^ The enzyme was effectively anchored using a well-known strategy:^31^ glutaraldehyde is employed as a coupling agent between the lysine residues and the amino groups brought on the silica surface by functionalisation with 3-aminopropyl-triethoxysilane (APTES). The role of APTES was shown to be decisive in the performance of these functional materials. Yet, neither the APTES functionalisation, nor the enzyme grafting were optimized.

In the present paper, we report significant improvements that can be obtained in the production of monolithic biocatalysts exhibiting enhanced activity. First, we inspect the spatial homogeneity of the APTES functionalisation and we propose a method leading to a more homogeneous distribution of the amino groups throughout the materials and subsequently to enhanced catalytic performance. Second, we address repeatability issues by controlling the water content during functionalisation. Third, we discuss the role of temperature during the enzyme grafting step. Finally, we consider the possibility to reduce the hydrolysable imine bonds formed in the covalent grafting process, to ensure a more robust immobilisation. We discuss the pertinence of these strategies in the context of flow biocatalysis.

## Experimental

### Materials

Acetone (≥99.9%), (3-aminopropyl)triethoxysilane (APTES; ≥ 98%), 4′-bromoacetophenone (BAP; ≥ 98%), *n*-dodecane (≥90%), hydrochloric acid (37% wt, aqueous solution), pyridoxal 5′-phosphate hydrate (PLP; ≥ 98%), sodium cyanoborohydride solution (5.0 M in 1 M NaOH; NaBH_3_CN), sodium hydroxide aqueous solution (50%), sodium pyruvate (≥99%), tetraethyl orthosilicate (TEOS; ≥ 98%), toluene (≥99.8%, anhydrous), trimethyltetradecylammonium bromide (TTAB; ≥ 99%) were purchased from Sigma-Aldrich. 4-Bromo-α-methylbenzylamine (BMBA; ≥ 99%; racemate), dibasic potassium phosphate (≥99%), dimethylsulfoxide (DMSO; ≥ 99.8%) and monobasic potassium phosphate (≥99%) were purchased from Acros Organics. d-Alanine (≥99%) was purchased from CarlRoth. Tetrahydrofuran (THF; ≥ 99.6%) was purchased from VWR Chemicals. Heat-shrinkable polytetrafluoroethylene (PTFE) tube was purchased from RS Components. Codexis generously supplied ATA-117 transaminase. Distilled water was applied for all synthesis and treatment processes.

### Silica monolith synthesis

Porous monoliths synthesis was performed according to Ungureanu *et al.*:^32^ 6 g of a concentrated hydrochloric acid solution (37% wt) were introduced in 16 g of a TTAB aqueous solution (35% wt). Then 5 g of TEOS was added. The aqueous phase was stirred until a monophasic hydrophilic medium was obtained. 35 g dodecane was then added dropwise while stirring to form an emulsion. The latter was cast into a polypropylene 10 mL flask, allowed to condense for 1 week at room temperature. Resulting material was washed three times with 50 mL of a THF/acetone mixture (1 : 1 v/v) (each washing lasted 24 h) and then gently dried in air during 3 days before calcined at 650 °C for 6 h (heating rate of 2 °C min^−1^ with a first plateau at 180 °C for 6 h). Porous monoliths were stored in a desiccator at room temperature.

### Monolith functionalisation

Grafted samples were denoted A*x*_Y_ where ‘A’ stands for APTES grafting, ‘*x*’ is the APTES concentration in the functionalisation solution (in mM), and ‘Y’ is the functionalisation method used for the monolith (method A, B, C or D).

#### Method A

2 monoliths (approximately 0.15 g each) were added into 25 mL of a toluene/APTES solution of desired concentration. Dynamic vacuum was applied to force the solution into the pores of the monoliths, until effervescence stopped. Static vacuum was then maintained for 24 h. Monoliths were separated from the toluene/APTES solution and washed three times with 25 mL of a toluene/acetone mixture (1 : 1 v/v). For each washing, wet monoliths were soaked into the washing solvents by applying dynamic vacuum until effervescence stopped and then static vacuum for 2 h. Functionalised monoliths were dried under vacuum at 60 °C for 24 h and then stored in a desiccator at room temperature.

#### Method B

This protocol is similar to “Method A”, but right after the effervescence stops, monoliths were removed from the grafting solution, and disposed on a grid in toluene-saturated atmosphere for 24 h (in a closed-desiccator containing a toluene flask for atmosphere saturation). APTES concentration used for this “dry impregnation” method was typically higher than in Method A. The monoliths were then washed, dried and stored in the same way as in Method A.

#### Method C

A re-hydroxylation protocol was applied (adapted from Zhuravlev)^33^ before functionalisation: monoliths were soaked in water at room temperature. Dynamic vacuum was applied until the effervescence stopped (to force the solution into the pores of the monolith). The wet monoliths were then inserted in a closed glass bottle, and maintained at 100 °C for 24 h. Monoliths were then dried at 190 °C for 24 h under vacuum on a glass Petri plate, and then cooled to room temperature (still in vacuum). Monoliths were then grafted with APTES following the procedure described in Method B.

#### Method D

The monoliths were dried at 120 °C for 24 h under vacuum before functionalisation. After cooling to room temperature (still under vacuum), monoliths were functionalised as in Method B, but using a water-saturated APTES solution.

### Characterisation

#### Attenuated total reflectance – infrared spectroscopy (ATR-FTIR)

Samples were analysed using a Bruker Equinox 55 with a Platinum ATR cell, with a diamond crystal, and Trans DTGS detector. 100 scans were taken for both background and samples, with a resolution of 2 cm^−1^. ATR correction was applied (number of ATR reflection is 1; angle of incidence is 45°; mean reflection index of sample is 1.5). Monoliths were systematically crushed, unless otherwise stated.

#### Thermogravimetric analysis (TGA)

Samples were dried under vacuum at 105 °C for 24 h. TGA was performed under air flow (50 mL min^−1^) with heating rate of 10 °C min^−1^ from 30 °C until 900 °C with a TGA/SDTA-851e Mettler Toledo equipment. Data analysis was performed using the STARe software.

#### Thermo-programmed water desorption

Crushed samples were analysed using a Catlab-PCS Hiden Analytical equipped with a QGA mass spectrometer. The argon flow rate was 30 mL min^−1^. Temperature program was: (i) plateau at 50 °C for 10 minutes, (ii) heating to 700 °C (heating rate 3 °C min^−1^), (iii) plateau at 700 °C for 10 minutes. Detected mass/charge ratios were: 17 and 18 (H_2_O), 28 (CO), 32 (O_2_), 40 (Ar) and 44 (CO_2_).

### Transamination reaction

#### Batch mode

**Fig. 1:**
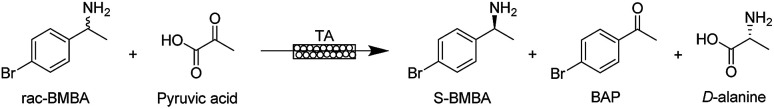
Model reaction: kinetic resolution of S-BMBA.

A model reaction was used to assess catalytic activity of ATA-117: the transamination of pyruvate with racemic BMBA, to produce BAP and d-alanine. As the enzyme only accepts the *R*-enantiomer of BMBA as a substrate, this reaction is a kinetic resolution (Fig. 1). Typical conditions were 30 °C, phosphate buffer 0.1 M pH 8, pyridoxal phosphate 2.02 mM, sodium pyruvate 10 mM, racemic BMBA 10 mM, DMSO 5%. Batch reactions were carried out in 5 mL round bottom glass flasks under moderate magnetic stirring.

#### Flow mode

A monolith was inserted into a heat-shrinkable PTFE tube, which perfectly fits the monolith shape when heated, preventing preferential flows along monolith sides. The heat-shrinkable PTFE tube was also shrunk around stainless steel male connectors (surrounded by PTFE tape to ensure sealing), themselves connected to PTFE pipes. This device was connected to a PP Terumo syringe used to deliver a controlled liquid flow. Monolithic reactors were placed in a thermo-regulated water bath (Fig. S1†). Each reactor was first impregnated with 50 mL of a 1% glutaraldehyde aqueous solution (flow rate of 1.4 mL min^−1^) at 4 °C or 30 °C. Then, the enzyme was grafted in the monolith by flowing 50 mL of the buffered transaminase solution (0.2 g L^−1^ in potassium phosphate buffer 0.1 M pH 8, pyridoxal phosphate 2.02 mM, sodium pyruvate 10 mM) with a flow rate of 1.4 mL min^−1^, with the water bath maintained at 4 °C or 30 °C. After, the reactor was washed at 30 °C with 50 mL buffer, with a flow rate of 1.4 mL min^−1^, until no enzyme is detected in the outflow. Practically, this was achieved after using approximately 30 mL of buffer solution. Sample denotation is based on the name of the monolith (functionalised following Method A, B, C, or D) with ‘GA’ as a suffix and TA as a prefix when the monolith has been loaded with glutaraldehyde and with the transaminase. An additional suffix ‘30d’ is added for the samples which have been loaded with the enzyme at 30 °C. The reaction medium (same conditions as in batch reactions) was loaded in the syringe and injected into the reactor with a flow rate of 0.11 mL min^−1^. Catalytic activity was determined by collecting the outflows and measuring BMBA consumption and BAP production in gas chromatography. During enzymatic reaction, actual flow rates were continuously monitored allowing an accurate determination of contact time.

Conversion was determined on 100 μL samples taken from the reaction medium. 10 μL of sodium hydroxide (2 M) was added and the mixture was vortexed for 1 second. 500 μL of dichloromethane was then added to the aqueous phase and vortexed for 10 seconds to allow extraction of BAP and BMBA into the organic phase. This extraction step was repeated twice and the organic phase was collected and analysed by gas chromatography (Bruker Scion 456 GC with a WCOT fused silica BR-5 column (30 m × 0.32 mm ID × 1.0 μm) and helium as carrier gas (25 mL min^−1^), oven temperature at 150 °C, split ratio of 20, injector temperature at 250 °C, flame ionisation detector temperature at 300 °C (air flow 300 mL min^−1^, H_2_ flow 30 mL min^−1^)). The yield is defined as the proportion of rac-BMBA converted into 4′-bromoacetophenone (BAP; in %). The maximum yield for the kinetic resolution is 50%.

#### Enzyme quantification

Enzymatic concentrations were assessed by a modified Bradford method,^34,35^ using bovine serum albumin protein as a standard. 500 μL of sample were added to 1500 μL of Bradford reagent. After 10 minutes incubation at room temperature, absorbance was read at 595 and 470 nm with a spectrophotometer ThermoScientific Genesys 10S-Vis. Enzyme loading in the monoliths was determined by assessing the difference of enzyme concentration in the inflows and the outflows. The immobilisation efficiency can be calculated as the ratio between the calculated loading and the amount of enzyme used in the grafting step (10 mg). It varied from a few percent to 20% maximum depending on the method used to functionalise the monoliths.

## Results and discussion

### Functionalisation (Method A) and APTES dispersion

In our recent work,^30^ we demonstrated the feasibility of immobilizing a transaminase (ATA-117, from Codexis) on macrocellular silica monoliths for the enantioselective transamination in continuous flow. The enzyme was covalently attached to the amino-functionalised silica monolith (with APTES), using glutaraldehyde as a coupling agent. In the best conditions, using a monolith functionalized according to the protocol here called “Method A” and with a contact time of 10 min, we obtained a stable BAP yield of 6%, with excellent enantiospecificity (only R-BMBA was converted).

**Fig. 2:**
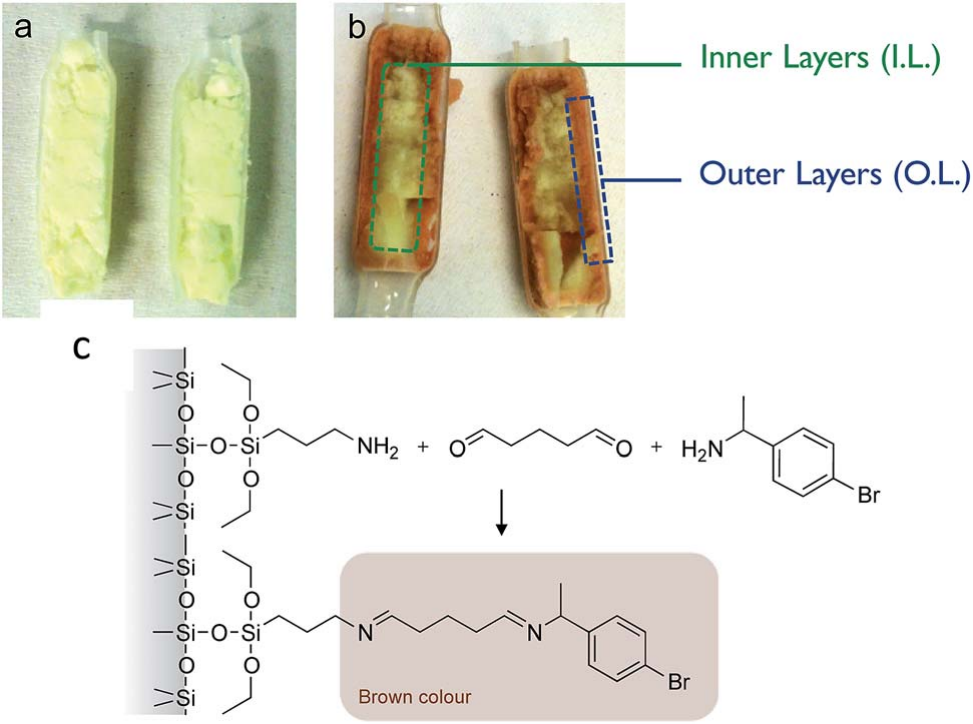
(a) Photograph of a monolith prepared *via* Method A but without APTES (blank) and cut lengthwise after the flow reaction (TA-A0_A_-GA), (b) photograph of the TA-A10_A_-GA cut lengthwise after the flow reaction. For subsequent characterisation, the outer layer (O.L.) was collected by scratching the monolith over 1 mm thickness. The inner layer (I.L.) was the core of the monolith. (c) Proposed reaction between the amino moiety and BMBA in the presence of glutaraldehyde, leading to the brown colour in the APTES-functionalised monoliths.

After the reaction, the monolith was cut lengthwise, and a brownish crust was observed (Fig. 2). This crust was not observed when a blank was used (no APTES in the functionalisation solution). This visually indicates that the APTES dispersion throughout the monolith is not homogeneous. We propose that this colour is linked to the reaction between the aminopropyl groups grafted in excess and the reactant BMBA in the presence of glutaraldehyde (Fig. 2c).

**Fig. 3:**
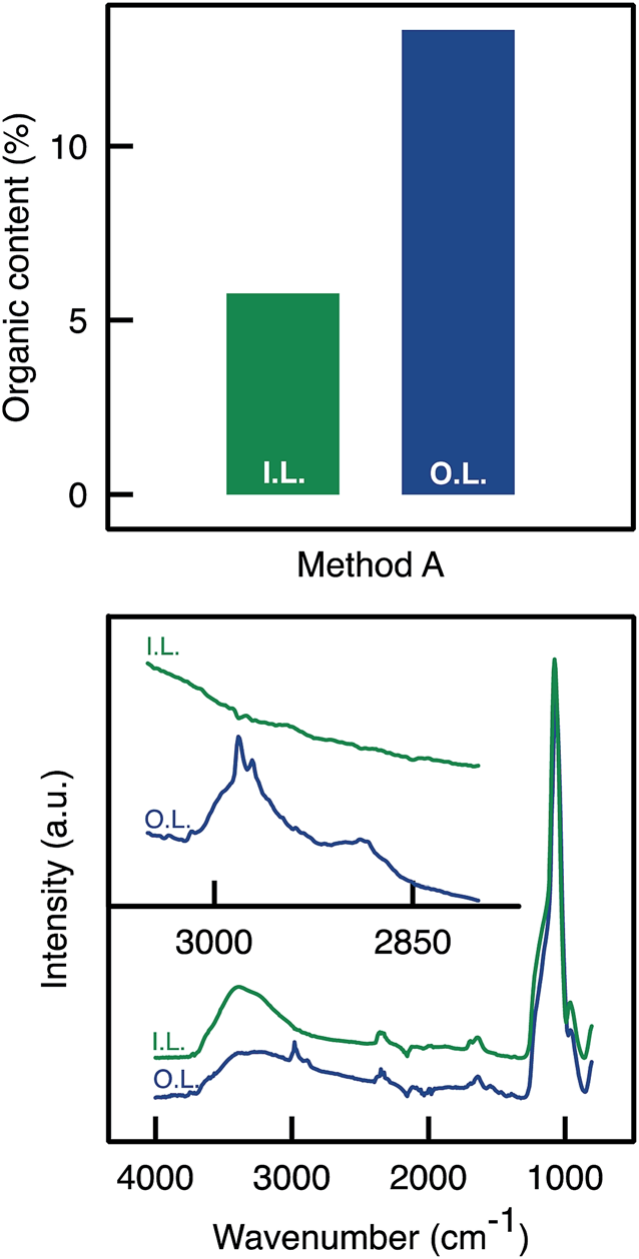
(Top) Total organic content determined by TGA analyses on the outer and inner parts of the A50_A_ monolith. (Bottom) C–H stretching band absorbance was detected around 2900 cm^−1^ only for the outer layer of the sample A50_A_.

ATR-FTIR and TGA analyses were performed on the inner and outer layers of the APTES functionalised monolith. TGA analyses (Fig. 3) confirmed an uneven APTES dispersion in the monolith functionalised by Method A. Clearly, the weight loss (associated to grafted aminopropyl moieties) was much higher from the sample scratched from the outer layers than from the inner layer. This was also confirmed by ATR-FTIR analyses as the C–H stretching band was detected around 2900 cm^−1^ only for the outer layer and not for the inner layer (Fig. 3).

Our interpretation of the heterogeneity of the APTES grafting was linked to the method of functionalisation. During the functionalisation process with Method A, the monolith was soaked into a large excess of the grafting solution. Thus, the porosity of the monolith was filled with an APTES solution of relatively low concentration, and the external environment was also composed of the same APTES solution. During the 24 h of functionalisation, APTES molecules could continuously diffuse from the excess solution and enter into the monolith, thereby functionalizing mostly the outer part of the monolith. In this way, a radial APTES gradient was established. It appears reasonable to put forward that a more homogeneous APTES functionalisation throughout the monolith would lead to a more effective enzyme immobilisation and possibly, to higher biocatalytic performance. This is why Methods B, C, and D were proposed as attempts to improve the quality of the APTES functionalisation step.

### Improving APTES-functionalisation

#### Dry impregnation (Method B)

**Fig. 4:**
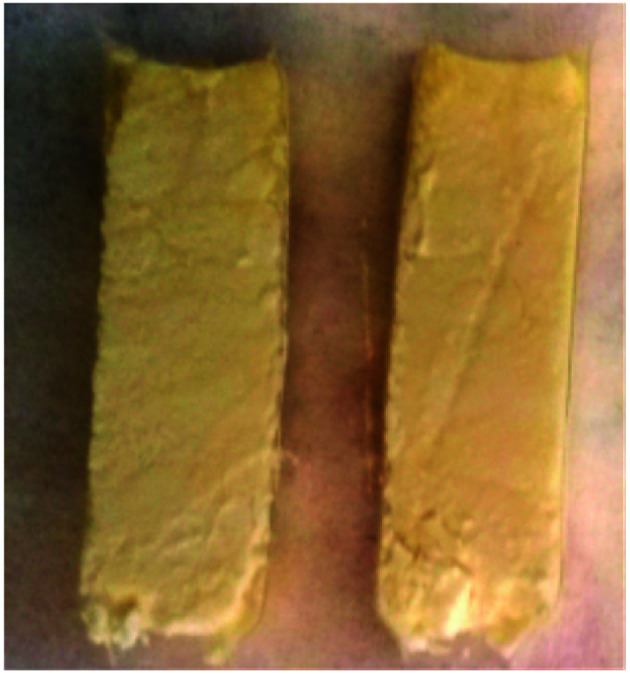
Functionalisation Method B led to a homogeneous APTES dispersion into monoliths (TA-A500_B_-GA sample).

**Fig. 5:**
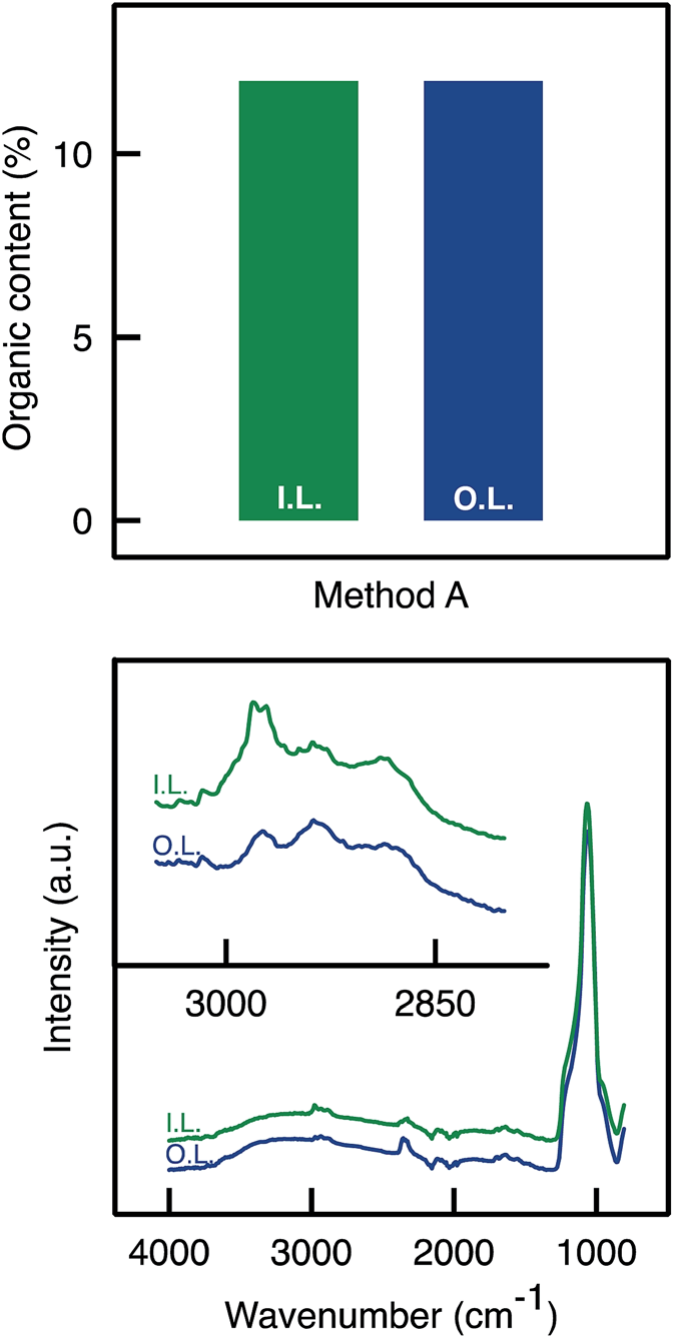
Characterisation of a monolith functionalised *via* Method B (A500_B_). (Top) TGA Analysis on the outer and inner layers (Method B allowed a homogeneous APTES dispersion; same organic content in both layers). (Bottom) ATR-FTIR: C–H stretching band absorbance was detected around 2900 cm^−1^ in both outer and inner layers with similar intensity.

In Method B the monoliths were briefly dipped in the grafting solution under vacuum and then removed from the solution. The solution was more concentrated (500 mM) in APTES than in Method A. The monoliths were then placed on a grid and maintained in a toluene-saturated atmosphere for 24 h. They then followed the same procedure for the washing, drying, storage, loading of glutaraldehyde, enzyme immobilisation, and then they were tested in the kinetic resolution of BMBA (*vide infra*). The monoliths were cut lengthwise after the catalytic reaction in flow. APTES dispersion appeared to be homogeneous throughout the monoliths as no crust was observed (Fig. 4). Instead, a homogeneous yellow colouring was observed throughout the monolith. In order to confirm the homogeneous APTES dispersion, ATR-FTIR and TGA analyses were performed on the inner and outer layers of the samples functionalised by Method B. TGA analyses (Fig. 5) showed that Method B achieved a homogeneous functionalisation throughout the monolith since the same organic content was measured in both layers. This was confirmed by ATR-FTIR analysis (Fig. 5) as the signal attributed to C–H stretching (2900 cm^−1^) was detected with similar intensity in both layers.

**Fig. 6:**
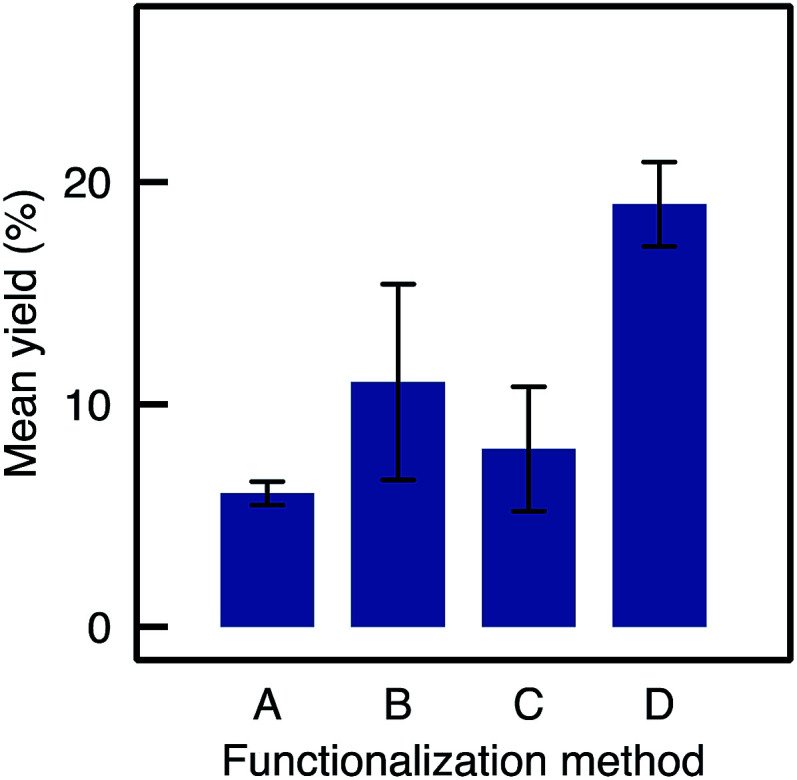
Mean yields and RSD for flow reactions depending on the functionalisation method used (samples are TA-A50_A_-GA, TA-A500_B_-GA, TA-A500_C_-GA, TA-A500_D_-GA).

Eight TA-A500_B_-GA monoliths were prepared independently following strictly the same procedure used to load the enzyme and were tested in the kinetic resolution of BMBA. The catalysts showed on average a yield of 11%, significantly higher than the 6% yield obtained with Method A samples (Fig. 6). A 2 mg loading was obtained in average for A500_B_-GA samples (standard deviation, RSD, was 57% for the loading measurement on the eight monoliths).

Thus, the better dispersion of APTES throughout the monoliths seems to be beneficial for the enzyme anchoring. However, the repeatability was poor (RSD was 40% for the final yield obtained with the eight independent monoliths prepared *via* Method B, see error bar in Fig. 6). This may be assigned to a relatively high variability of the silanol content on Si(HIPE) monoliths from batch-to-batch. Indeed, according to Zhuravlev,^33^ dehydroxylation of the silica surface should be expected to occur during monolith calcination. In contrast, water from air humidity could slowly re-hydroxylate the silica surface before the functionalisation procedure is applied. This rehydroxylation step was not controlled in Method B and may have varied from one experiment to the other. Yet, those silanols are the anchoring points for the grafting of the aminopropyl groups. Thus, Method C was implemented in order to increase the silanol surface density and to control the rehydroxylation step.

#### Rehydroxylation prior to silanisation (Method C)

**Fig. 7:**
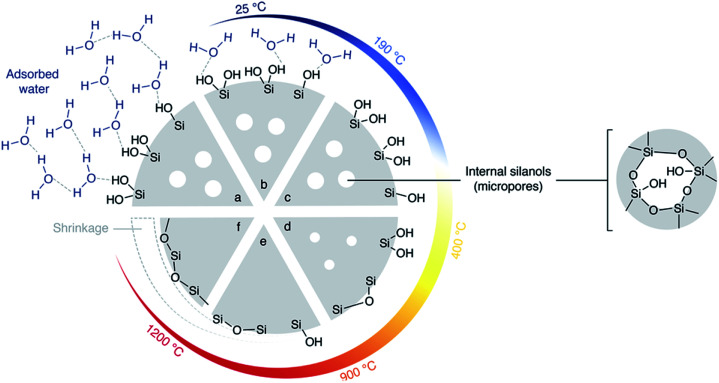
Silica heated under vacuum: Zhuravlev model.^33^ (a) Hydrated silica at 25 °C. Many silanols are present at the surface as well as internal silanols (in micropores). Water is in excess and forms multilayers. When heated under vacuum (b) silica gradually loses surface water. (c) At the key temperature of 190 °C, silica is totally dehydrated but still contains many silanols. (d) Beyond 190 °C, silanols condensation into siloxane bridges gradually occurs at the surface. Over 400 °C, this condensation is irreversible. (e) Over 900 °C, condensation internal silanols and condensation of most of the surface silanols occurs. (f) Finally, by heating in vacuum up to 1200 °C, silica surface loses its last silanols into siloxanes.

Si(HIPE) monoliths re-hydroxylation was inspired by Zhuravlev^33^ (Fig. 7): after soaking in water, monoliths were heated at 100 °C in a closed glass recipient. The monoliths were then dried at 190 °C under vacuum, in order to remove excess water, while maintaining a large amount of hydroxyl groups. Temperature-programmed desorption of water has been performed on rehydroxylated samples. Water desorption is followed by mass spectrometry when the samples are gradually heated under argon atmosphere. Above 400 °C, water desorption is assigned to silanol condensation. A higher amount of water was shown to desorb from the rehydroxylated sample, confirming that the rehydroxylation procedure indeed allowed creating more silanol groups at the silica surface (Fig. S2†).

Benefiting from a higher surface density of hydroxyl groups, it was expected that more APTES molecules could be grafted. One the one hand, TGA analyses confirmed the presence of a larger organic content on the samples that have been rehydroxylated (A500_C_*versus*A500_B_, see Fig. S3†). One other hand, ATR-FTIR analyses did not allow evidencing a higher intensity for organic moieties in the sample prepared by Method C as compared to Method B (not shown).

In the flow mode transamination reaction, the TA-A500_C_-GA biocatalysts showed a slightly lower yield (8.4%) as compared to A500_B_-GA (Fig. 6). The method still showed a low repeatability (RSD on the yield was 35% when three different monoliths functionalised by Method C were tested independently). Thus, although Method C seems to allow increasing the silanol surface density on the silica monoliths, it did not allow to increase the efficiency of the APTES functionalisation, and the biocatalytic performance obtained after subsequent enzyme grafting were not improved either. Moreover, the reproducibility on the final yield remained poor.

#### Controlling water availability (Method D)

The presence of water during silanisation reactions is known to be a critical parameter. Indeed, a little amount of water is needed to catalyse the silanisation reaction.^36^ Yet, the presence of higher amounts of water is known to provoke the polymerisation of APTES molecules leading to a non-uniform APTES distribution that could explain the variability in the biocatalytic reaction.^37,38^ Regarding the functionalisation of the silica monoliths, it was expected that these highly porous solids adsorb water (from ambient humid air) after the calcination step. This was identified as a possible cause for a lack of reproducibility in the functionalisation step. Also, dried toluene used for APTES grafting could absorb variable water amounts from ambient humidity, as soon as the bottle was opened. Thus, Method D was applied as an attempt to strictly control the presence of water throughout the functionalisation process.

On the one hand, Si(HIPE) monoliths were dried at 120 °C under vacuum for 24 h in order to remove excess water from the monolith. After cooling under vacuum, functionalisation of the monolith was applied without further delay. On the second hand, the APTES functionalisation solution was prepared using water-saturated toluene as the solvent, so that the water input was strictly the same for all samples. The preparation was repeated independently on three different silica monoliths.

**Fig. 8:**
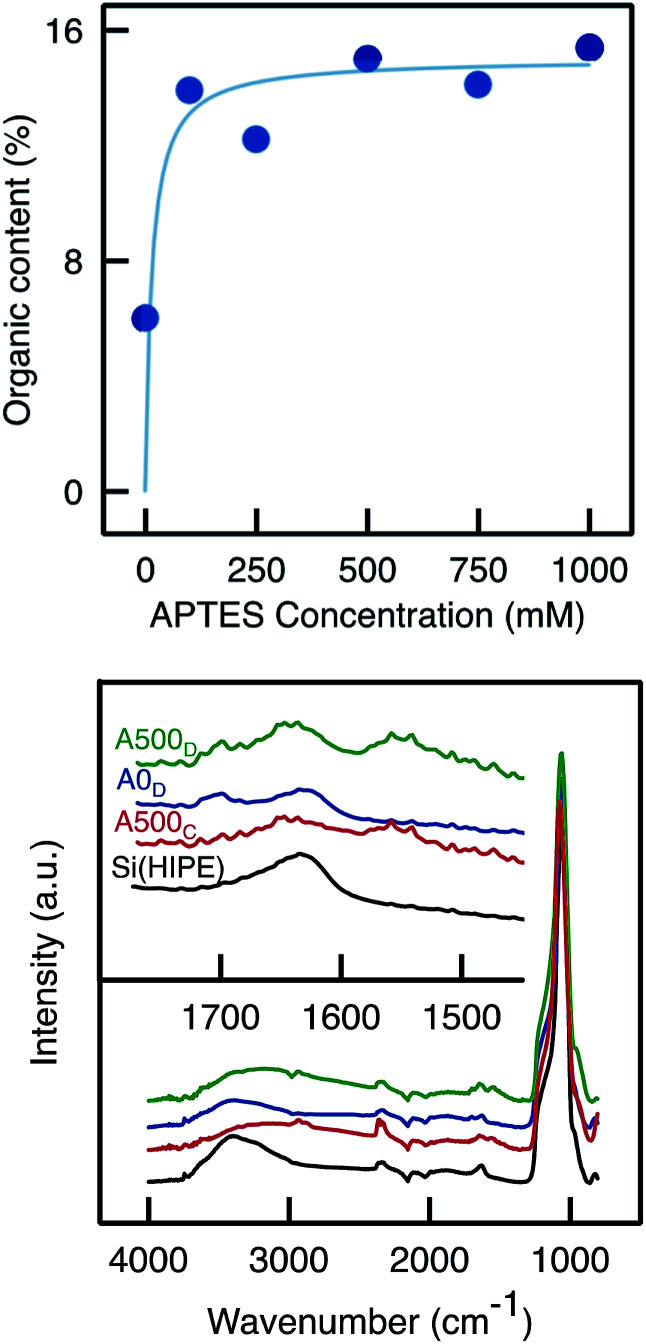
(Top) Total organic content as measured by TGA on monoliths functionalised *via* Method D with APTES solution of various concentration. (Bottom) ATR-FTIR spectra of Si(HIPE), A500_C_, A0_D_ (blank) and A500_D_.

**Fig. 9:**
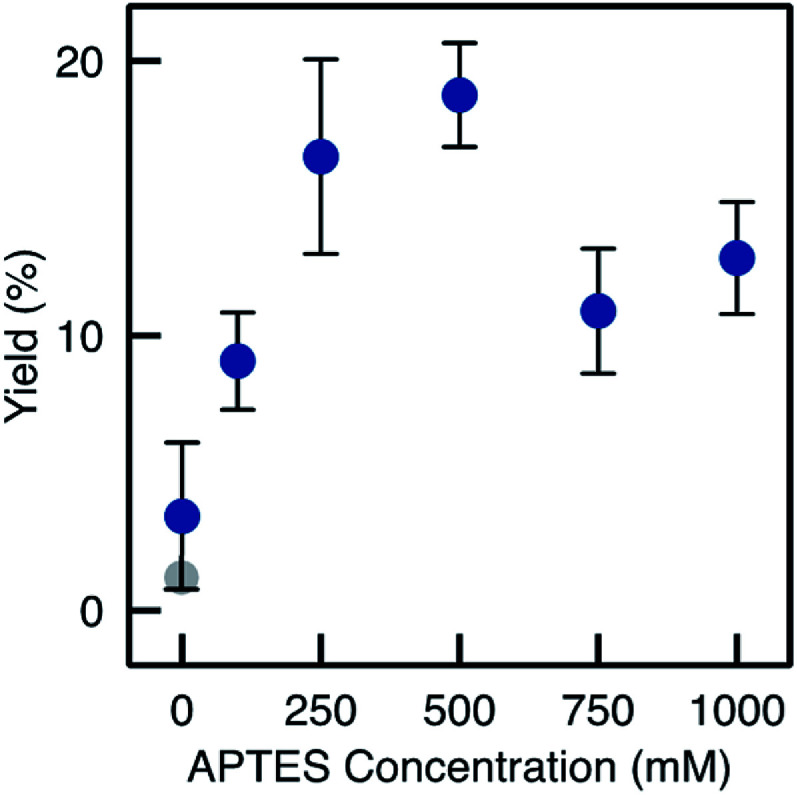
BAP yields with the monoliths prepared by method D (A*x*_D_, *x* = 0 to 1000 mM). The grey dot represents the yield and loading for TA-Si(HIPE) sample. Error bars correspond to the RSD on three independent experiments with freshly prepared monoliths for each APTES concentration.

TGA analyses of samples functionalised with APTES by Method D confirmed that the organic content was higher than in the blank sample (which has undergone the same functionalisation procedure but without APTES; Fig. 8). However, the organic content did not increase markedly with the APTES concentration and instead seemed to level off, suggesting that the monoliths are saturated, already upon impregnation with 100 mM APTES solution. The organic content was similar as that obtained with Method C (∼15%). ATR-FTIR analyses (Fig. 9) showed a slight increase in C–H vibration band absorbance with the presence of APTES. Moreover, similar to Method C, the primary amine scissoring band appeared (at 1570 cm^−1^)^39^ with APTES functionalisation.

Despite relatively low enzyme loading (0.7 mg per monolith maximum, see Fig. S4†), the biocatalysts prepared from monoliths functionalised *via* Method D showed high performance. The yield reached 19% on average with a much improved repeatability (*e.g*. RSD on the final yield was 9.2% for three different TA-A500_D_-GA monoliths, see Fig. 6). Yields increased quite linearly with the APTES concentration in the functionalisation solution, up to 250 mM (Fig. 9). At higher APTES concentration, the catalytic yield dropped. This trend was related to the loading of enzyme that is immobilised, depending on the APTES concentration (Fig. S4†). The loading also reached a maximum for TA-A500_D_-GA and then decreased. From these measurement, it appears interesting to notice that the enzyme loading was high both for the pristine TA-Si(HIPE) sample and for the TA-A0_D_-GA (*i.e.* the blank without APTES). This suggests that the enzyme was immobilized in large amounts by simple adsorption, but that it was markedly deactivated upon such simple physisorption. Indeed, it is well documented that enzyme conformation – crucial for catalytic properties – could be altered during simple adsorption.^12,13^ In contrast, APTES grafting is suggested to confer a favourable environment to preserve enzyme activity (even if the total loading is lower). Hydrophobic effects brought by APTES grafting have been already highlighted in the literature to help preventing the deformation of the structure.^40^

When comparing the yields obtained with monoliths grafted by Method C and D, we observed that Method D allowed a higher yield as A500_C_ and A500_D_ exhibit respectively 8.4% and 19% yield. Thus, despite similar results in IR and TGA, Method D provides monoliths with higher biocatalytic activity. This may be due to a better control on the APTES dispersion throughout the monolith with Method D. This confirms the crucial role of water in such silanisation reaction. With this method in hand, it is possible to obtain macrocellular monoliths, which exhibit much higher biocatalytic yield, together with a highly improved repeatability. An APTES concentration of 250–500 mM was identified as the best compromise to ensure that enough organic moieties are grafted and to optimize both the enzyme loading and the catalytic yield in the flow reaction. Table 1 summarizes the main improvements obtained by adapting the functionalisation method.

**Table 1:** Functionalisation improvements – from Method A to D

Sample	Dispersion	Mean yield^b^ (RSD)	Mean organic content^a^	Estimated enzyme loading
A50_A_	Poor	6% (9%)	11%	1.2 mg
A500_B_	Good	11% (40%)	7%	2.0 mg
A500_C_	Good	8% (35%)	12%	1.7 mg
A500_D_	Good	19% (9.2%)	15%	0.7 mg

a TGA on entire functionalised monolith (before enzyme loading).

b RSD is calculated based on three independent experiments carried out on freshly prepared monoliths (except for A500_B_ for which eight independent experiments were carried out and used to calculate the yield and RSD).

### Improving the enzyme immobilisation efficiency

#### Effect of temperature

**Fig. 10:**
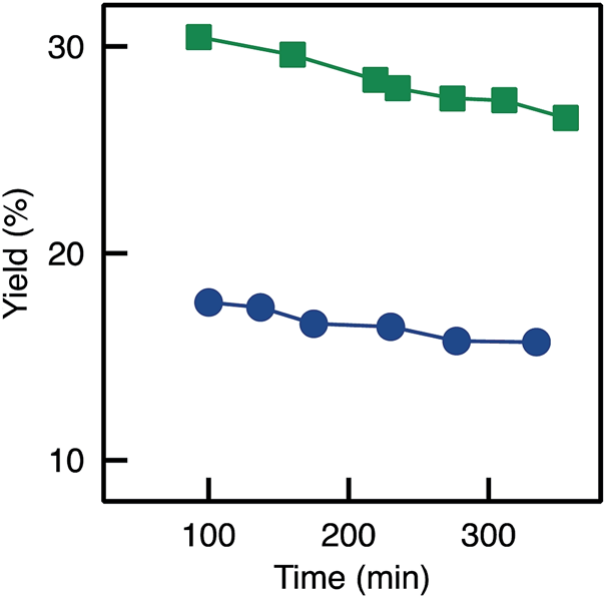
Evolution of the BAP yield with time for TA-A250_D_-GA-30d: (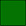) reference (no reduction); (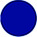) reduction with NaBH_3_CN (Red3).

Classically, enzymes are manipulated in cold conditions to prevent natural deactivation (*e.g.* due to residual protease activity).^41^ Accordingly, in the present work enzyme immobilisation was initially carried out at 4 °C. However, the grafting occurred as covalent bonds were created between the enzyme and the functionalised support, and therefore, temperature may play an important role. Thus we have also performed the same immobilisation procedure on the A250_D_ monolith at 30 °C (sample denoted TA-A250_D_-GA-30d). In this case, the catalytic performance multiplied by two, as the yield reached ∼30% (Fig. 10).

This result may find two explanations. First, the kinetics of the reactions that led to the grafting of the enzyme (imine bonds formation between the lysine and the aldehyde) was probably enhanced at higher temperature, leading to a more efficient grafting. Indeed, a higher enzyme loading was measured for TA-A250_D_-GA-30d (0.7 mg) as compared to the TA-A250_D_-GA (0.2 mg). Second, the lower activity obtained after immobilisation at 4 °C can tentatively be explained by the so-called “cold dissociation” phenomenon.^42^ Indeed, it is important to note that transaminases are homo-dimeric enzymes, with the active site formed only when two monomers assemble (Fig. S5†).^43^ According to Privalov,^42^ under low temperatures, some multimeric enzymes may be dissociated to the monomers. In this case, a higher proportion of the inactive monomers would have been immobilized on the monolith instead of the active dimers. Once two monomers are immobilized on different grafting sites, reforming the active dimer is impossible. Moreover, the immobilisation of monomer could have occurred through the lysine residue located on the active site, thereby locking the enzyme with the active site towards the support surface, blocking the access for the reactants. Thus, it is clear that enzyme grafting should be done at 30 °C instead of 4 °C and we suggest that further optimisation of the grafting temperature could lead to further increase in the performance.

#### Imines reduction

A slight deactivation was observed over time: TA-A250_D_-GA-30d samples exhibited 85% of its initial activity after 300 minutes on stream (Fig. 10). This can be an indication of a progressive deactivation of the immobilized enzymes or of a slow leaching of the immobilized enzymes from the monolith surface (yet, experimentally, no enzyme activity was detected in the outflows). In fact, the imine bond formed during enzyme anchoring (Fig. S6†) can in principle be hydrolysed, leading to enzyme detachment. A classical strategy to avoid such reaction is to reduce the imine to the amine, using for example sodium cyanoborohydride (NaBH_3_CN) as a reducing agent.^11,31,44,45^

In a first attempt (Red1), the reducing agent was injected right after the enzyme immobilisation (after washing). In this case, a poor activity level was obtained as compared to the reference experiment without reduction (Fig. S7†). As already reported for PLP-dependent enzymes,^46,47^ deactivation by cyanoborohydride was suspected. In a control batch reaction, we indeed observed that NaBH_3_CN strongly deactivates the transaminases (Fig. S8†). It should be noted that the first step of the transamination reaction mechanism (Fig. S9†) is the formation of an imine between the aldehyde group of the cofactor (PLP) and the essential primary amine of the enzyme active site (from a lysine residue). The reducing agent could therefore attack the imine bond, leading to the irreversible formation of a secondary amine, permanently locking the cofactor into the active site, and leading to deactivation.

In a second attempt (Red2) the transaminase solution (containing PLP in the buffer) was injected through the reactor for enzyme immobilisation, and then, the PLP was washed prior to the reduction with NaBH_3_CN. By doing so, it was hypothesized that the locking of PLP into the active site could be minimized. Before starting the reaction by injecting the reactants together with PLP, the reducing agent was washed. However, this procedure also led to poor yield (Fig. S7†). It can be put forward that PLP remained in the vicinity of the active site, despite the washing step.

Looking back at the batch experiments (Fig. S8†), we observed that the reaction medium could act as a protective agent for the active site: no deactivation occurred when the reducing agent was added directly in the reaction medium. One possible explanation is that the reactants (having a strong affinity for the active site and being present in relatively high concentrations, well above the *K*_M_) tend to protect the active sites simply by keeping them less accessible for the reducing agent. Thus, in a last attempt to reduce imine bond while keeping the biocatalysts active (Red3), the reducing agent was fed to the flow reactor during the flow transamination. This system showed some activity (Fig. S8†); yet, the yield was still much lower than for the reference sample (no reduction). Thus, the negative impact of the reducing agent on the active sites was still present in this case. In relative terms, however, the reduction (Red3) could allow to very slightly decrease the deactivation on stream since the decrease in yield as a function of time was slightly slower (Fig. 10).

## Conclusions

Starting with the proof-of-concept for carrying flow mode transamination reactions in a silica macrocellular monoliths loaded with transaminase enzymes, we here identify the essential parameters which allow reaching high catalytic performance; (i) functionalisation mode (dry *versus* wet), (ii) water availability during the functionalisation process and (iii) immobilisation temperature. In practice, briefly dipping the monolith in the functionalisation solution followed by aging in a solvent-saturated atmosphere allowed to obtain a homogeneous APTES dispersion throughout the entire monolith (confirmed by IR and TGA). Total water control during the process (by using dried monoliths and water-saturated solvent) allowed to minimize the batch-to-batch variability which affects enzyme immobilisation and biocatalysts efficiency. An attempt to reduce the imine bond formed upon enzyme immobilisation with APTES and glutaraldehyde was performed in order to minimize any putative enzyme leaching. It appeared that transaminases were irreversibly deactivated by the reducing agent, which disqualifies this strategy. Finally, we highlighted a strong beneficial effect of carrying out the immobilisation at 30 °C instead of 4 °C. This immobilisation temperature could advantageously be optimized in a future study. All in all, the optimized method presented here allowed to reach a 5-fold increase in activity for the studied transamination reaction.

## Conflicts of interest

There are no conflicts to declare.

## Supplementary Material

RA-009-C9RA02433F-s001
