# Supplementary material for: Biocatalytic transamination in a monolithic flow reactor: improving enzyme grafting for enhanced performance
Source: RSC Adv. 2019 Jun 12;9(32):18538–46. doi: 10.1039/c9ra02433f (PMC9064773; doi:10.1039/c9ra02433f)
Supplement: RA-009-C9RA02433F-s001 [file RA-009-C9RA02433F-s001.pdf]

Electronic Supplementary Material (ESI) for RSC Advances.
This journal is © The Royal Society of Chemistry 2019

Biocatalytic transamination in a monolithic flow reactor: improving enzyme grafting for enhanced performance

Electronic Supplementary Information

Ludivine van den Biggelaar, [a] Patrice Soumillion[b] and Damien Debecker[a,*]

[a] Institute of Condensed Matter and Nanosciences, UCLouvain, Place Louis Pasteur 1, 1348 Louvain-la-Neuve, Belgium. damien.debecker@uclouvain.be
[b] Louvain Institute of Biomolecular Science and Technology, UCLouvain, Place Croix du Sud 2, 1348 Louvain-la-Neuve; Belgium.

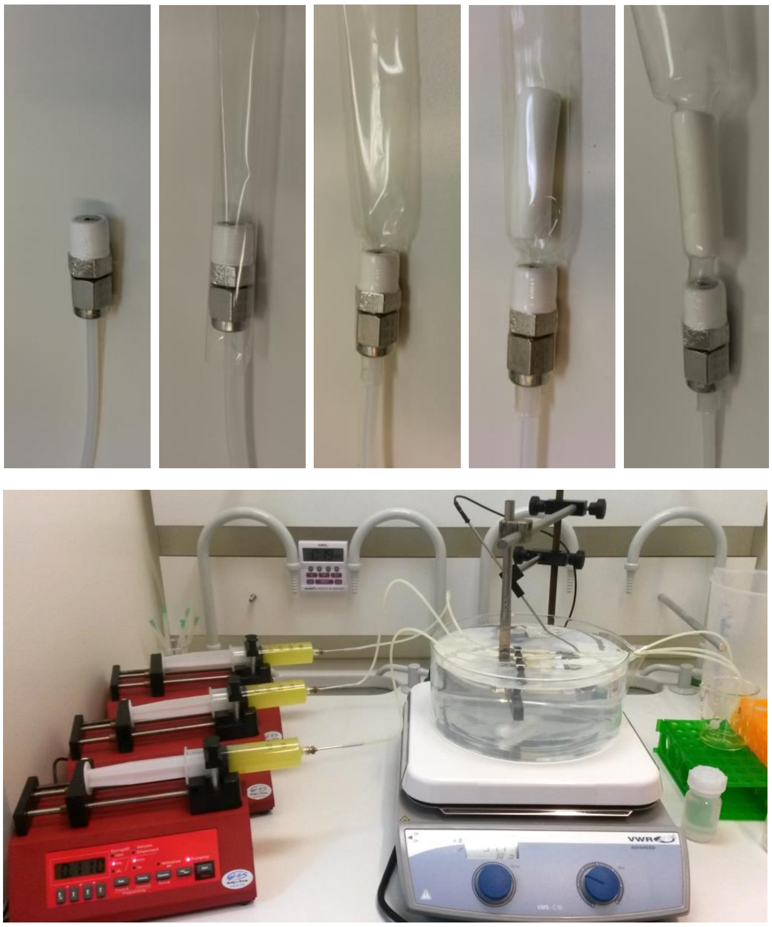

Figure S1 – (top) Picture depicting the casting of the monolith into the heat-shrinkable Teflon tube and connexion wit the PTFE tubing. (bottom) Picture of the experimental set-up for flow reaction. Monolithic reactors are connected to syringe-pumps and outflow collectors via PTFE tubes. Monoliths are and immerged in a temperature-controlled water bath.

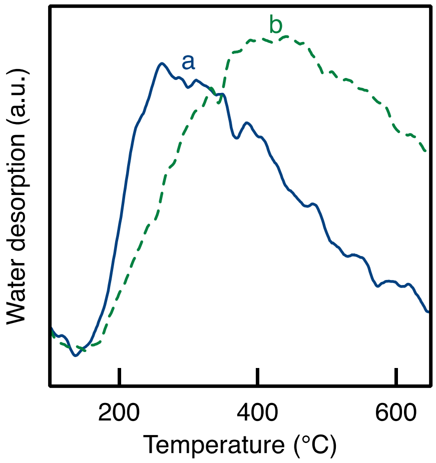

Figure S2 – Thermo programmed water desorption of (a) Si(HIPE) and (b) rehydroxylated Si(HIPE).

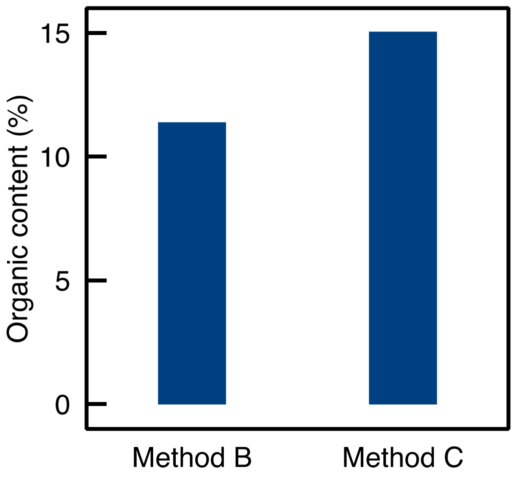

Figure S3 – TGA analyses of A500B and A500C samples. More organic functions were detected on the rehydroxylated samples. This suggested that the rehydroxylation process allowed featuring more silanols at the surface.

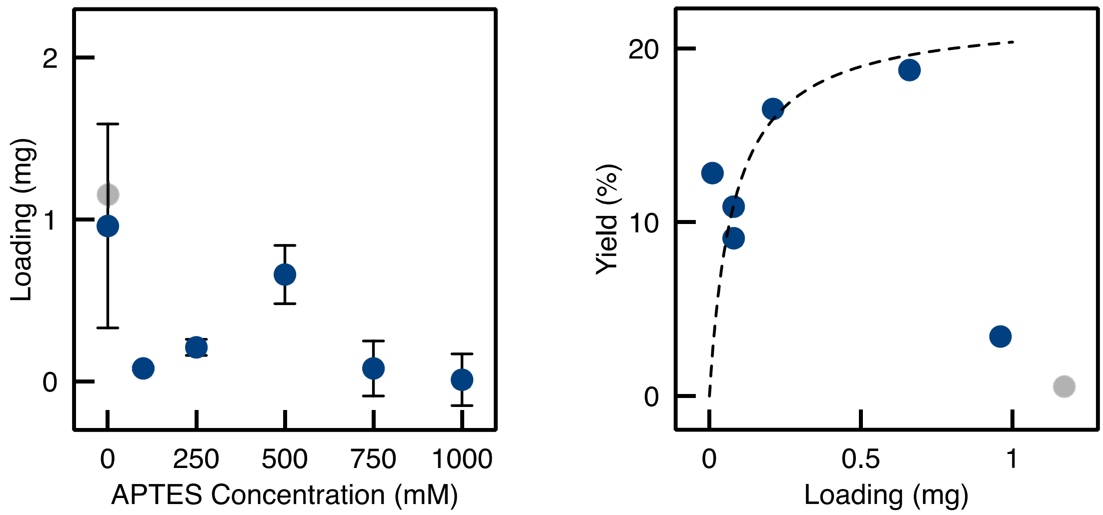

Figure S4 – (Left) Enzyme loading as a function of the APTES concentration used for functionalization in Method D. (Right) BAP yield as a function of the enzyme loading for the samples prepared by Method D. Dotted curve is only a guide to the eye. Shaded dots (●) represents the yield and loading for TA-Si(HIPE) sample

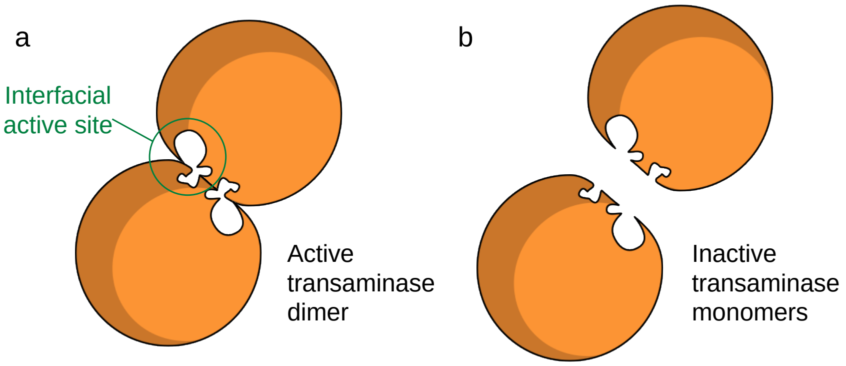

Figure S5 – Transaminases are dimeric enzymes. (a) Active sites are located on the interfacial area. (b) When the environment gets colder, monomers are suspected to undergo a reversible dissociation, leading to a "cold dissociation".[1]

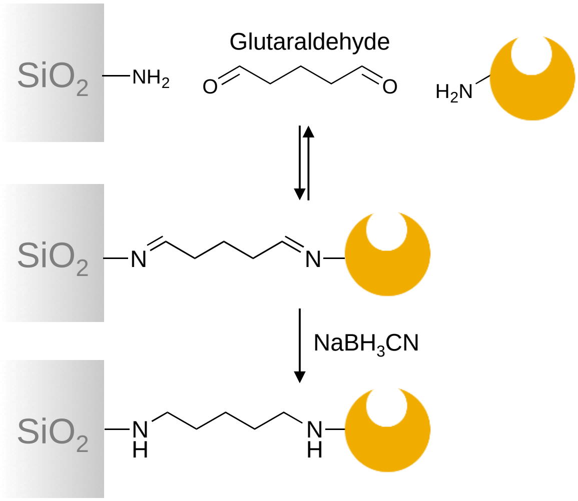

Figure S6 – Transaminases are immobilized through a reversible covalent bound (amines activated with glutaraldehyde, forming imines). Reducing imines into secondary amines lead to irreversible bonds.

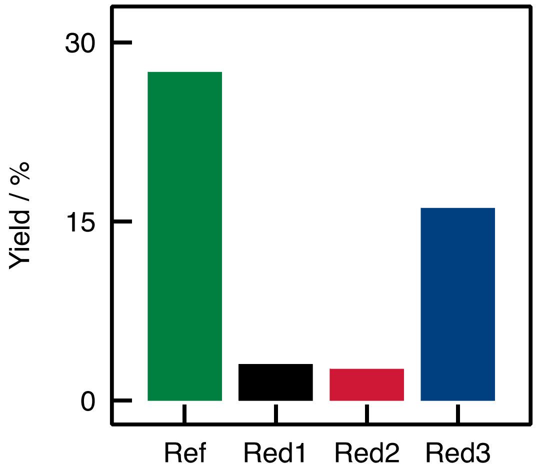

Figure S7 – Effect of imines reduction on A250D-30d samples yields. “Ref” stands for reference sample (no reduction).

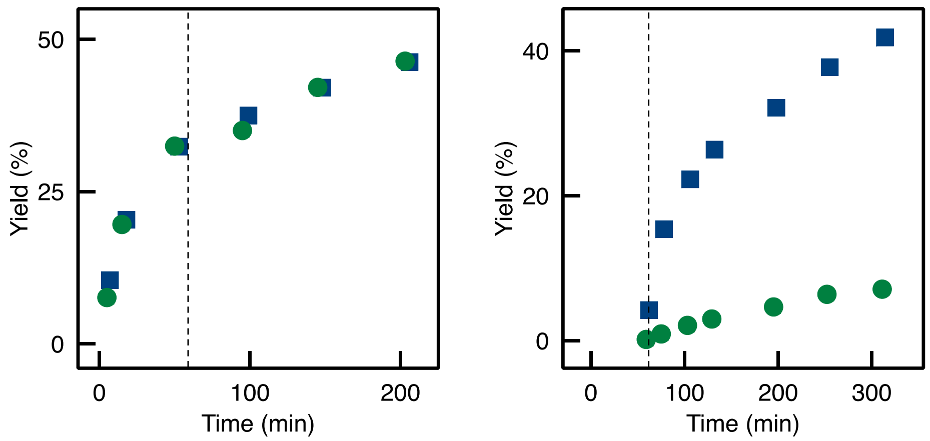

Figure S8 – (Left) Effect of the addition of $NaBH_3CN$ during the transamination in batch reactors (1h after starting the reaction, dotted line): (■) blank (no addition of $NaBH_3CN$); (●) addition of $NaBH_3CN$. Reactions conditions: 10 mM rac-BMBA, 10 mM pyruvate, 0.5 g/L PLP, 4.4 µM ATA-117, 5 % DMSO, $NaBH_3CN$ 50 mM, 30 °C. No deactivation occurred in batch reactors when reducing agent was added after 1 hour reaction. (Right) Effect of pre-incubation with 50 mM $NaBH_3CN$ on transamination reactions in batch reactors (1 hour pre-incubation, dotted line): (■) blank (no pre-incubation with $NaBH_3CN$); (●) one hour pre-incubation with $NaBH_3CN$. Reactions conditions: 10 mM rac-BMBA, 10 mM pyruvate, 0.5 g/L PLP, 4.4 µM ATA-117, 5 % DMSO, 30 °C. When the reducing agent was added to transaminases solutions prior to the batch reaction (one hour pre-incubation before the transamination reaction), a strong deactivation was observed.

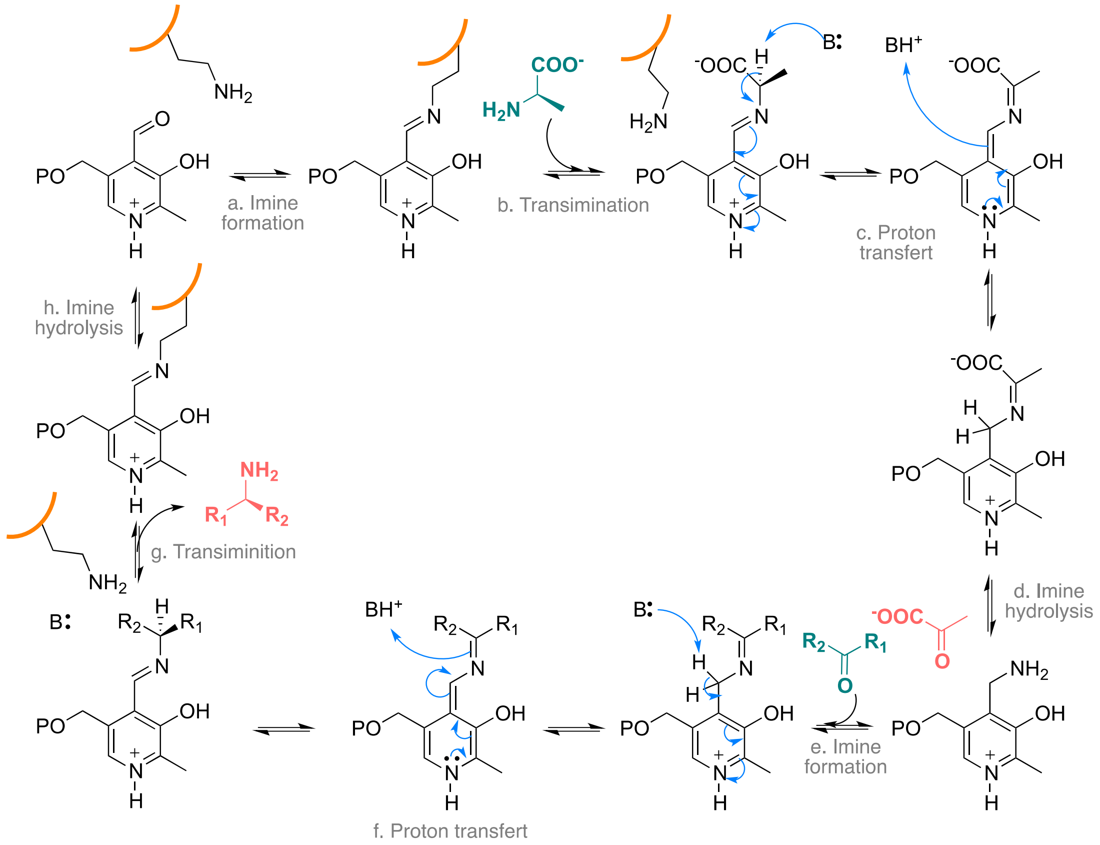

Figure S9 – Transamination mechanism. Two half-reactions execute the ping-pong bi bi mechanism: from (a) to (d), the amine reacts with the PLP cofactor to form the ketone by-product and a PLP amine-derivative (PMP, Pyridoxamine phosphate); from (e) to (h), the ketone reacts with the PMP to form the amine and release the PLP. PO stands for phosphate, and B for basic molecule or residue. Adapted from referecences[2-4].

References

1. P. L. Privalov, *Crit. Rev. Biochem. Mol. Biol.*, 1990, **25**, 281-306.
2. D. Koszelewski, K. Tauber, K. Faber and W. Kroutil, *Trends Biotechnol.*, 2010, **28**, 324–332.
3. K. E. Cassimjee, B. Manta and F. Himo, *Org. Biomol. Chem.*, 2015, **13**, 8453–8464.
4. K. E. Cassimjee, M. S. Humble, V. Miceli, C. G. Colomina and P. Berglund, *ACS Catal.*, 2011, **1**, 1051–1055.
